# Comorbidities in frontal fibrosing alopecia and lichen planopilaris: A systematic review and meta-analysis

**DOI:** 10.1016/j.jdin.2026.08.014

**Published:** 2026-09-17

**Authors:** Juan Luis Sanz-Cabanillas, Francisco José Gómez-García, Elena Beatriz Sanz-Cabanillas, Jesús Gay-Mimbrera, Beatriz Isla-Tejera, Antonio José Vélez García-Nieto, Juan Ruano

**Affiliations:** a Inflammatory Immune-Mediated Chronic Skin Diseases Laboratory, IMIBIC/University of Córdoba, Córdoba, Spain; b Department of Dermatology, Reina Sofía University Hospital, Córdoba, Spain; c Department of Pharmacy, Reina Sofía University Hospital, Córdoba, Spain

**Keywords:** autoimmune diseases, comorbidities, frontal fibrosing alopecia, lichen planopilaris, meta-analysis, odds ratio, systematic review, systemic lupus erythematosus, thyroid diseases

*To the Editor:* Frontal fibrosing alopecia (FFA) and lichen planopilaris (LPP) are related lymphocytic cicatricial alopecias traditionally considered part of the same disease spectrum.^1^ However, accumulating clinical, histopathologic, transcriptomic, phenotypic, and epidemiologic evidence increasingly supports distinguishing them as overlapping but biologically distinct entities.^2^^,^^3^ We therefore conducted a systematic review and meta-analysis to characterize the spectrum of comorbidities associated with FFA and LPP and to explore potential differences between the two phenotypes.

MEDLINE (PubMed) and Embase were searched from inception through June 18, 2025. The protocol was registered in PROSPERO (CRD420250656067), and reporting followed PRISMA 2020 recommendations. Eligible studies were case-control studies reporting comparator-based comorbidity data for FFA or LPP. Two reviewers independently screened studies, extracted data, and assessed methodological quality using the Joanna Briggs Institute Critical Appraisal Checklist for case-control studies. Random-effects meta-analyses of log odds ratios (ORs) were performed for comorbidities reported in at least 2 independent studies. Exploratory mixed-effects meta-regression analyses compared pooled associations between FFA and LPP. Given shared control populations in some studies, the limited number of eligible comparisons and the absence of significant interactions after false discovery rate correction, these analyses were considered hypothesis-generating. Detailed methods are provided in the Supplementary Material, available via Mendeley at https://data.mendeley.com/datasets/6bc7bzzrvh/1.

Twenty-three case-control studies were included, comprising 1814 patients with FFA and 6656 with LPP, and more than 65 million controls. In FFA (Fig 1, *A*), the strongest and most consistent associations were observed for systemic lupus erythematosus (OR 4.34, 95% confidence interval [CI] 1.92-9.78; k = 7; I^2^ = 0%), vitiligo (4.06, 1.55-10.60; k = 6; I^2^ = 12.1%), thyroid disease (1.78, 1.22-2.59; k = 4; I^2^ = 0%), hypothyroidism (1.76, 1.26-2.45; k = 5; I^2^ = 0%), allergic contact dermatitis (3.19, 2.32-4.37; k = 2; I^2^ = 0%), and rosacea (2.29, 1.28-4.08; k = 4; I^2^ = 17.7%). Lichen planus pigmentosus showed a large but imprecise and non-significant association (4.33, 0.54-34.66).

**Fig 1:**
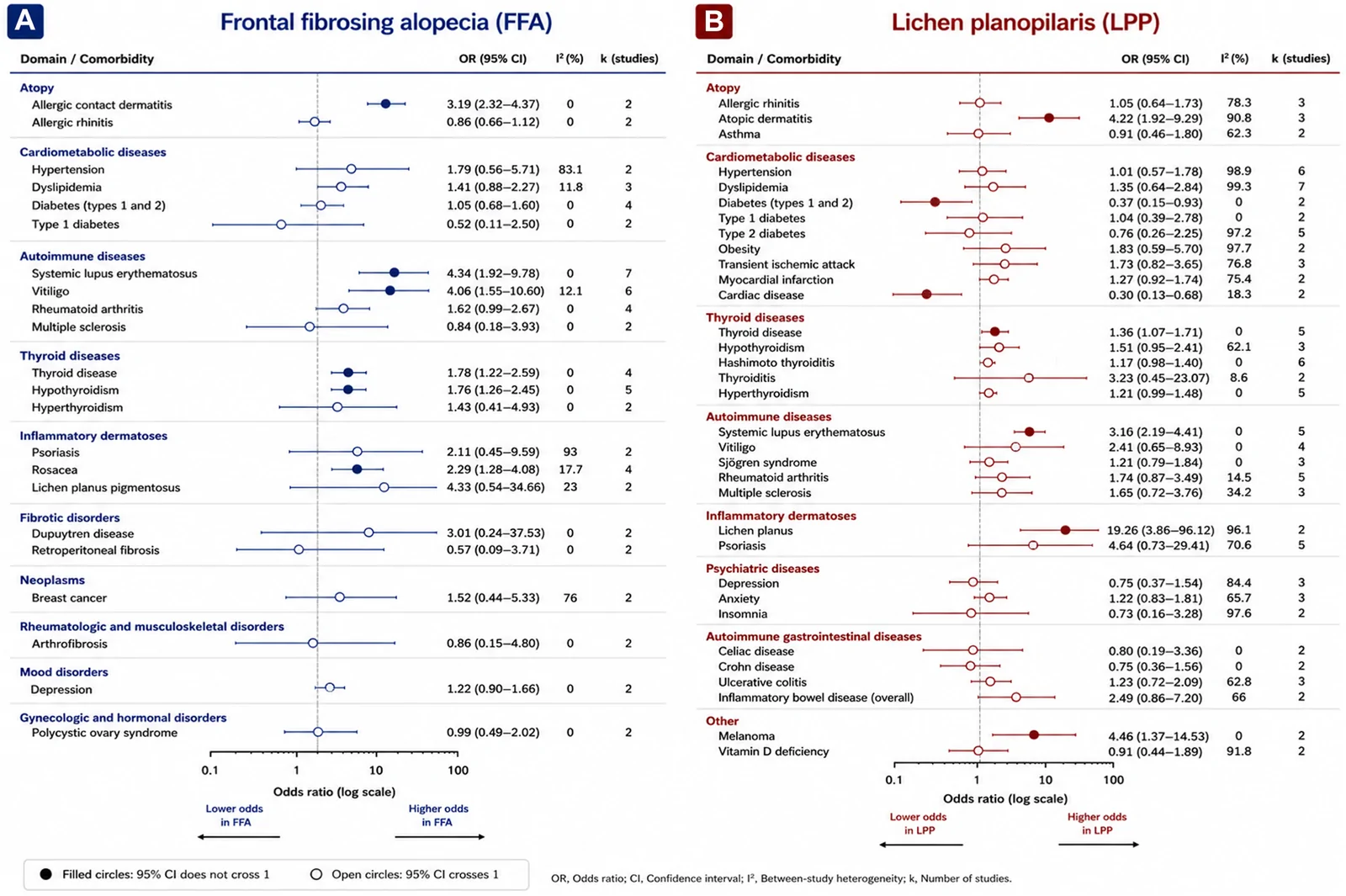
Comparator-based comorbidity associations in frontal fibrosing alopecia and lichen planopilaris. **A,** Frontal fibrosing alopecia (FFA). **B,** Lichen planopilaris (LPP). Pooled odds ratios (ORs) and 95% confidence intervals (CIs) from random-effects meta-analyses are shown on a logarithmic scale. Comorbidities are grouped by clinical domain. Filled circles indicate statistically significant associations (95% CI not crossing 1), whereas open circles indicate non-significant associations (95% CI crossing 1). I^2^ denotes between-study heterogeneity, and *k* the number of contributing studies.

In LPP (Fig 1, *B*), systemic lupus erythematosus was also associated (3.10, 2.19-4.41; k = 5; I^2^ = 0%), whereas thyroid disease showed a more modest association (1.36, 1.07-1.71; k = 2; I^2^ = 0%). Additional associations included atopic dermatitis (4.22, 1.92-9.29; k = 3; I^2^ = 90.8%), lichen planus (19.26, 3.86-96.12; k = 2; I^2^ = 96.1%), and melanoma (4.46, 1.37-14.53; k = 2; I^2^ = 0%). Overall, FFA and LPP shared several important autoimmune and inflammatory comorbidity associations. Exploratory interaction analyses did not identify any statistically robust differences between the two phenotypes after false discovery rate correction (Supplementary Table VIII, available via Mendeley at https://data.mendeley.com/datasets/6bc7bzzrvh/1), although nominal differences were observed for selected comorbidities.

Taken together, these findings broadly align with emerging transcriptomic and epidemiologic evidence suggesting partially distinct immune, stromal, immunometabolic, and demographic patterns across primary lymphocytic cicatricial alopecia subtypes.^2^^,^^3^ Nevertheless, further adequately powered comparative studies are required before phenotype-specific comorbidity profiles can be established. Clinical application should remain limited to history-taking, symptom-directed examination, and individualized laboratory or multidisciplinary evaluation rather than universal screening. Limitations include observational study designs, heterogeneous comorbidity definitions, residual confounding, the use of shared control populations in some studies contributing to both FFA and LPP estimates, the limited number of studies contributing to several interaction analyses, and the exploratory nature of these comparisons.

### Declaration of generative AI and AI-assisted technologies in the writing process

During the preparation of this work, the authors used ChatGPT (OpenAI) for language editing and code troubleshooting under author supervision. Following use of this tool, the authors critically reviewed, edited, and verified all content and take full responsibility for the final manuscript.

## Conflicts of interest

None disclosed.
